# Challenging the Status Quo to Shape Food Systems Transformation from a Nutritional and Food Security Perspective

**DOI:** 10.3390/foods11040604

**Published:** 2022-02-20

**Authors:** António Raposo, Renata Puppin Zandonadi, Raquel Braz Assunção Botelho

**Affiliations:** 1 CBIOS (Research Center for Biosciences and Health Technologies), Universidade Lusófona de Humanidades e Tecnologias, Campo Grande 376, 1749-024 Lisboa, Portugal; 2 Department of Nutrition, Faculty of Health Sciences, University of Brasilia, Brasilia 70910-900, Brazil; renatapz@unb.br (R.P.Z.); raquelbotelho@unb.br (R.B.A.B.)

**Keywords:** consumer, environment, food policy, food production, food safety, food security, nutrition, sustainability

Food security and nutrition have been prominent elements of the international development agenda. Over time, however, development priorities and challenges have oscillated, and the investment required has not been sustained. A broader consensus has emerged: one that guarantees food security and, in all its aspects, reduces hunger and malnutrition to promote strong economies, human and planetary health, and sustainable development. Our moral imperative is to positively change food systems to ensure that the food we produce is accessible, sustainable, safe, healthy, and equitable for everyone. Therefore, this special issue about Food Systems and Nutritional and Food Security focuses on connecting the importance of food systems to change nutrition and food security around the globe.

In this context, attitude and knowledge of health, food, and nutrition can be one of the keys to facing food insecurity in several countries. Health and nutritional education are imperative to fight malnutrition [1,2,3,4]. Knowledge in these aspects is essential in tackling misinformation and promoting good food choices. Since birth, it can be constructed along life using households, schools, the internet, and other channels to allow learning on food, health, and nutrition and stimulate adequate food choices [1,2,3,4,5,6,7].

Adequate and healthy diets imply choosing and consuming balanced and adequate foods on nutrients and amount, variety, and sustainable aspects. Therefore, achieving the main social, economic, environmental, cultural, and security goals [1,3,6,7,8,9,10,11,12,13,14,15,16]. Food production must also be alert to the waste produced in the food chain related to environmental pollution and food waste that could be used to feed food-insecure people [10,15,17]. In order to support sustainable and healthy diets and food security, it is also essential to search for alternatives to stimulate local production and regional food consumption. Other priorities are reducing animal suffering and meat consumption, food production in sufficient quantity and quality with the least possible waste production, and understanding the main aspects of food, nutrition, society, and the environment [11,12,13,14,15,16,17,18,19,20,21,22].

During these past two years of the COVID-19 pandemic, the world has faced new food security and safety challenges. Firstly, food sales worldwide became more complex, and farmers faced many overstocked products leading to food loss [8]. On the other hand, countries’ economic crisis conducted to more unemployment, therefore, more food insecurity. Governments need to learn from this period and improve the food supply chain with innovative techniques and logistics to have more organized food systems [8]. To guarantee the four pillars of food security, availability, access, stability, and utilization, Sun and Zhang [9] emphasize the importance of trade openness and sustainable food system strategies. Guiné et al. [7] discuss that the agrifood supply chain should be improved through policies worldwide to promote access to healthy and sustainable food. Authors also call the attention to researchers to rethink the four pillars of food security to include new dimensions like climate change.
